# How to treat jatrogenic ureteral injury after posterior spinal surgery? Case report and review of literature

**DOI:** 10.1002/iju5.12612

**Published:** 2023-07-26

**Authors:** Petar Kavaric, Aleksandar Magdelinic, Almir Rebronja, Marko Albijanic, Eldin Sabovic, Nenad Radovic, Marko Vukovic

**Affiliations:** ^1^ Department of Urology Clinical Centre of Montenegro Podgorica Montenegro

**Keywords:** iatrogenic injury, posterior spine surgery, ureter

## Abstract

**Introduction:**

Entry into the retroperitoneal space during open posterior spinal surgery introduces the rare possibility of iatrogenic ureteral injury.

**Case presentation:**

We describe a case of ureteral injury after spinal surgery in a 49‐year‐old female with persistent lumbar pain and high fever 2 weeks after spinal surgery. After admission to the urology department, a computer tomography scan was performed and revealed right‐side hydronephrosis grade III and large retroperitoneal fluid collection. After radiological confirmation of right ureteral injury, a ureteral stent was placed, but 4 weeks later, ureteral stricture was confirmed on antegrade pyelography. Therefore, surgical ureteroplasty was indicated 2 months after initial admission to the urology department. Six weeks later, the stent was removed, and intravenous pyelography revealed a normal ureteral passage.

**Conclusion:**

There should be a low threshold for ureteral injuries after spinal cord surgery in patients with high fever and elevated blood creatinine levels.

**Abbreviations & Acronyms**
CT: computer tomography
TLDF: transforaminal lumbar discectomy and foraminotomy

**Keynote message**

After spinal cord surgery, one should have a low threshold for ureteral injuries, and surgical treatment might be considered immediately after an unsuccessful trial with ureteral stent placement.

## Introduction

Although traditional spinal fusion surgery is associated with more complication reports than a minimally invasive approach, ureteral injuries are still very rare.^1^, ^2^, ^3^, ^4^ However, in these rare circumstances, medicolegal implications may be involved in addition to devastating consequences.^4^ Here, we present a case of a 49‐year‐old female with CT urography and retrograde pyelography confirmed ureteral injury after TLDF. In this case report, we aim to elucidate the vulnerability of the ureter during posterior lumbar surgery.

## Case report

**Fig. 1:**
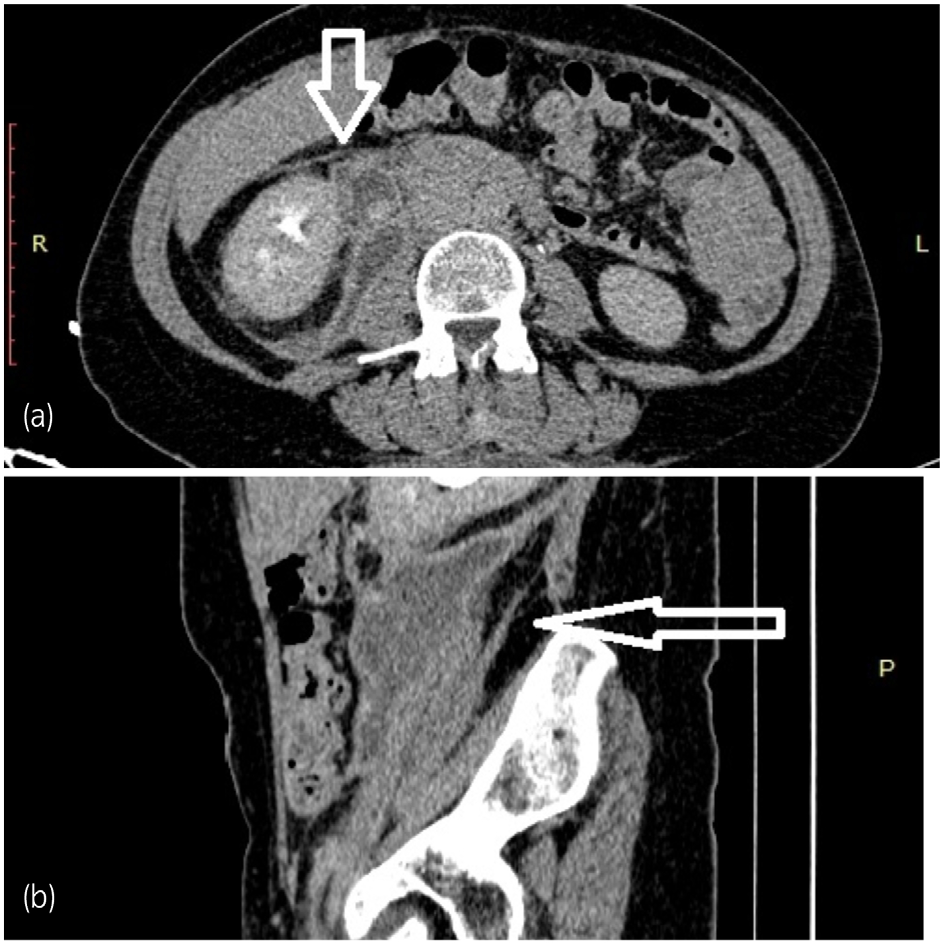
CT urography revealed urinary extravasation from right ureter (a) transversal plane; (b) sagittal plane. Arrow points out the urinoma formation.

**Fig. 2:**
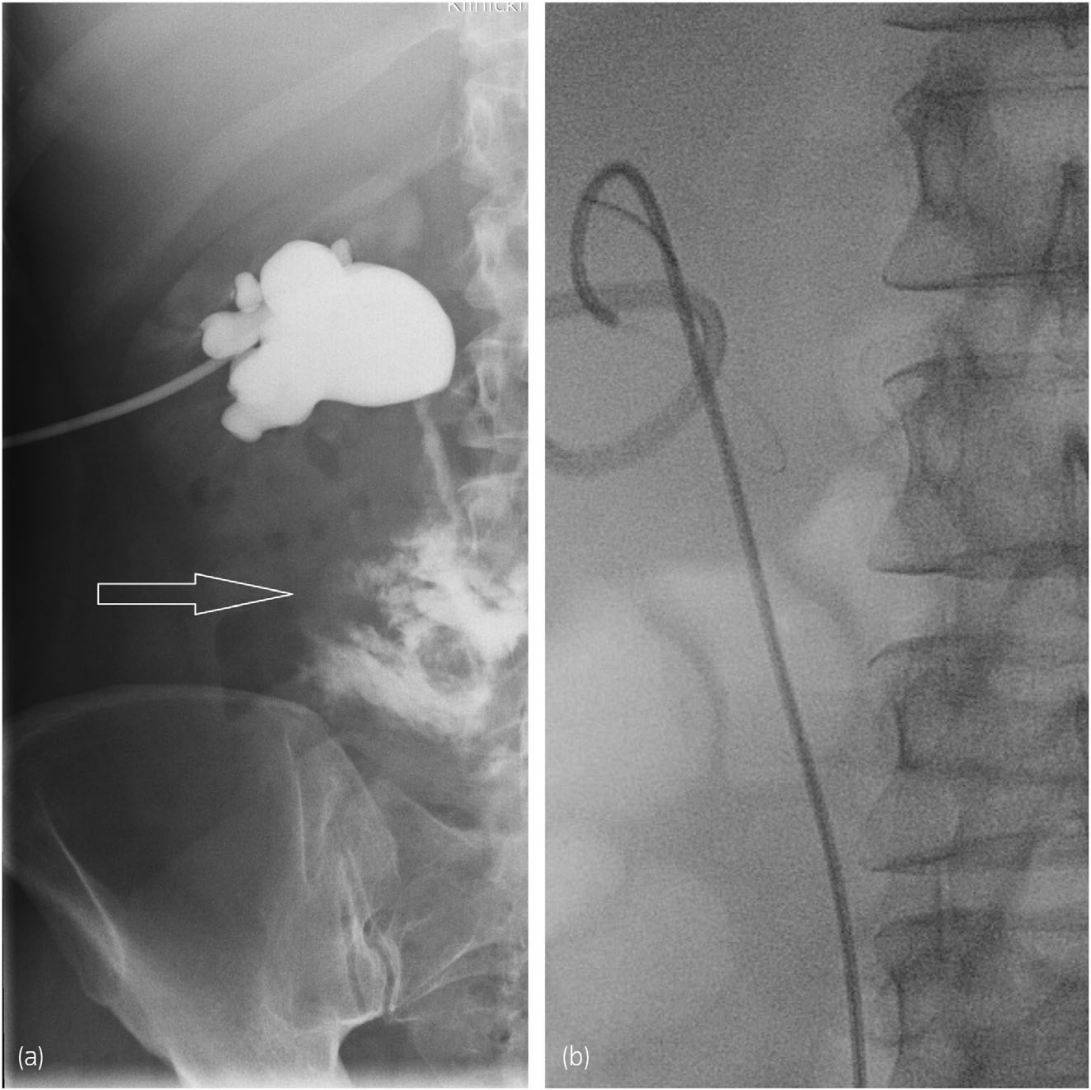
(a) Antegrade pyelography confirmed the proximal ureteral injury. Arrow points‐out contrast extravasation; (b) placement and position of Double‐J stent in right kidney and ureter.

A 49‐year‐old female presented to our department complaining of severe lumbar pain and a high fever. Her past medical history revealed a neurosurgical procedure for discus hernia performed 2 weeks prior (TLDF). There was no report about other medical histories, previous traumatic incidents, or radiation exposure. After admission to the urology department, a CT scan was performed and revealed grade III right‐side hydronephrosis and large retroperitoneal fluid collection starting from the right renal pelvis and propagating caudally and laterally, with a diameter of 11 × 6 cm. CT urography showed evidence of urinary extravasation from the right ureter (Fig. 1). The nephrostomy was placed subsequently, and right antegrade pyelography confirmed proximal ureteral injury (Fig. 2a). Furthermore, a cystoscopy was performed, and a ureteral stent was placed on the right side (Fig. 2b).

**Fig. 3:**
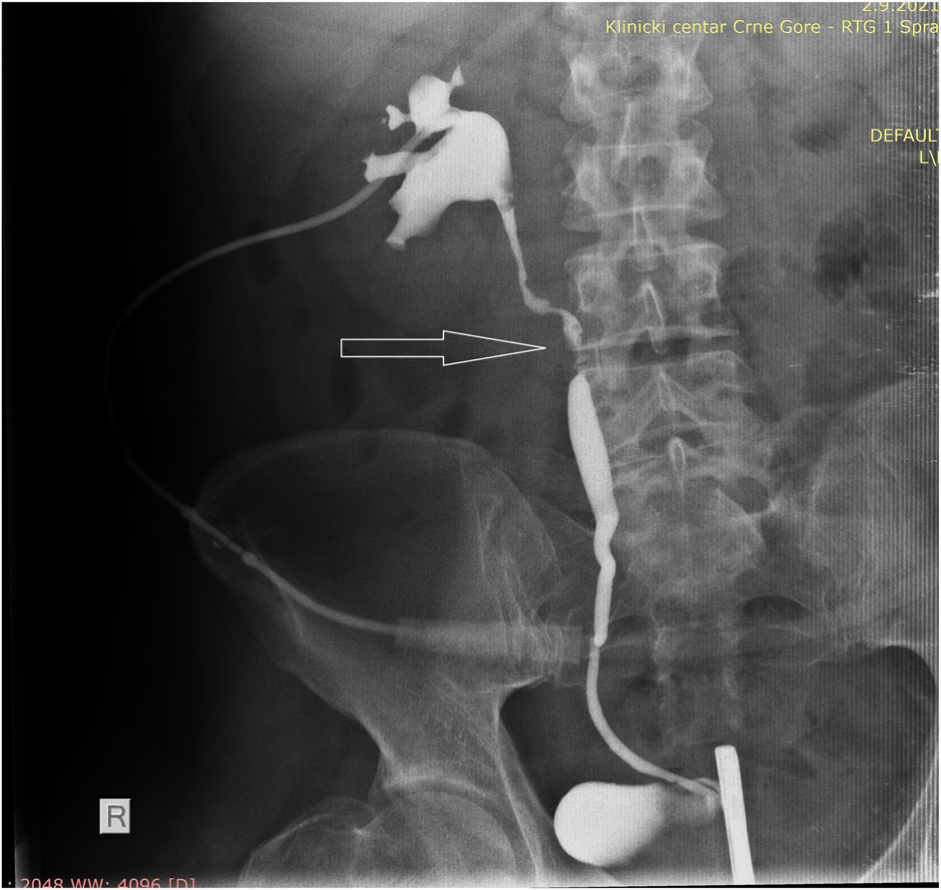
Combine antegrade and retrograde pyelography with the detection of ureteral stricture location (arrow).

**Fig. 4:**
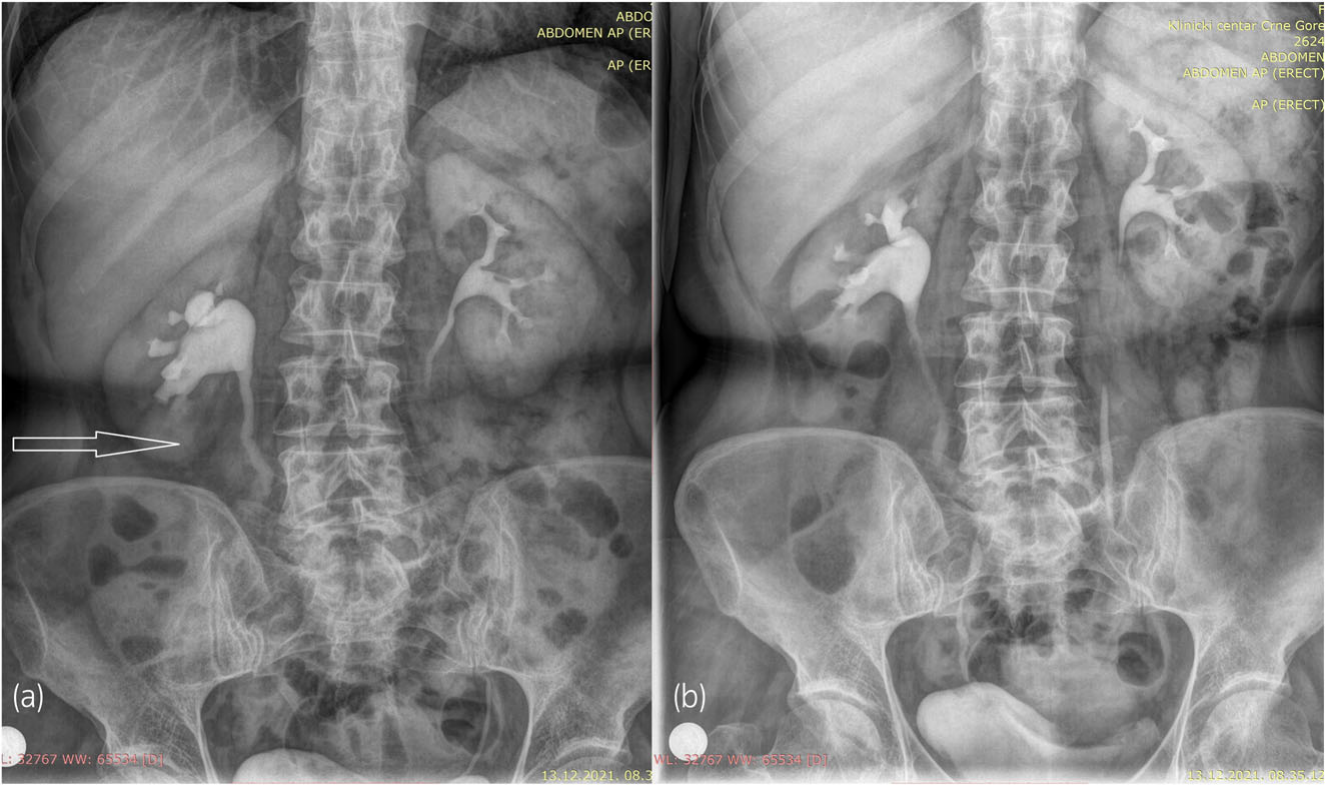
Intravenous pyelography after reconstructive ureteral surgery. Normal ureteral passage of the right proximal (a) and distal ureter (b) without residual stricture.

Four weeks later, the ureteral stent was removed, and control antegrade pyelography was performed (Fig. 3). Since complete ureteral stricture persisted during follow‐up, open surgical ureteroplasty was indicated 2 months after initial admission to the urology department. We resected approximately 15 mm of the stenotic part of the lumbar ureteral segment and performed a primary end‐to‐end ureteral anastomosis over the ureteral stent. Six weeks later, the stent was removed, and intravenous pyelography revealed a normal ureteral passage (Fig. 4). The patient continued to do well 6 months postoperatively, with no further complications.

## Discussion

Ureteral injury is a rare but serious potential complication of spinal procedures. It is often clinically unsuspected, as symptoms are nonspecific, and the patient may present weeks and even months after the injury.^5^ It should be considered in the differential diagnosis of any patient who presents with symptoms of acute abdominal pain after lumbar spine surgery.

In this case, we reported a right ureteral injury sustained in a female patient after TDLF. We suspected that the patient had delayed abdominal and lumbar pain associated with a fever due to a ureteral injury. When planning the treatment of ureteral injury, it is recommended to have a highly sensitive diagnostic tool to determine the exact location and diameter of the ureteral injury.^6^ Therefore, we ordered and diagnosed ureteral injury using contrast‐enhanced CT since the symptoms were nonspecific and occurred several weeks after the surgery.^1^ Although several treatment options are possible, upon injury detection, surgical treatment is often inevitable and sometimes requires a complex reconstruction technique.^6^ If possible, a one‐stage ureteral injury repair operation should be performed to prevent ureteral stricture.^7^ A minimally invasive approach is favorable but sometimes highly challenging, especially in neglected cases.^2^ In this case, we were able to perform open end‐to‐end ureteral alignment (ureteroureterostomy), which was successful due to the short stricture length and favorable stricture location.

## Conclusion

There should be a low threshold for ureteral injuries after spinal cord surgery in patients with symptoms of delayed lumbar or abdominal pain associated with a high fever and elevated blood creatinine levels. Contrast‐enhanced CT and retrograde urography are recommended in the diagnosis of ureteral injury, and open surgery with stricture resection and possible primary anastomosis is preferable after a previous unsuccessful trial with ureteral stent placement.

## Author contributions

Petar Kavaric: Conceptualization; investigation; visualization. Aleksandar Magdelinic: Formal analysis; funding acquisition; software; validation. Marko Albijanic: Data curation; software; validation. Almir Rebronja: Investigation; methodology; project administration. Eldin Sabovic: Data curation; validation; visualization. Nenad Radovic: Project administration; supervision; visualization; writing – review and editing. Marko Vukovic: Investigation; methodology; writing – original draft.

## Conflict of interest

The authors declare no conflict of interest. No part of this paper has been presented, published, or submitted for publication elsewhere in this or in any other language. All procedures for animal handling and treatments were made in accordance with the principles of the Declaration of Helsinki from the World Medical Association.

## Approval of the research protocol by an Institutional Reviewer Board

No ethical approval was required for this case report.

## Informed consent

Verbal informed consent has been obtained and is documented in the medical record.

## Registry and the Registration No. of the study/trial

Not applicable.
